# Assessment of the microbiological quality of fresh produce on sale in Sicily, Italy: preliminary results

**DOI:** 10.1186/s40709-015-0026-3

**Published:** 2015-02-28

**Authors:** Cinzia Cardamone, Aurora Aleo, Caterina Mammina, Giuseppa Oliveri, Anna Maria Di Noto

**Affiliations:** Istituto Zooprofilattico Sperimentale della Sicilia “A. Mirri”, via G. Marinuzzi 3, 90129 Palermo, Italy; Department of Science for Health Promotion and Mother-Child Care “G. D’Alessandro”, University of Palermo, via del Vespro 133, 90127 Palermo, Italy

**Keywords:** Fresh produce, Vegetables, Food safety, Hygiene quality, Foodborne pathogens

## Abstract

**Background:**

Fresh produce occupies an increasingly important place in the human food supply because of its health-promoting nutritional properties. Most fresh produce is eaten raw or after minimal processing and, consequently, pathogen contamination can represent a serious health risk. There has been an increase in foodborne outbreaks and cases associated with fresh produce, but literature data about the prevalence of pathogen contamination are inconsistent. This study was undertaken to assess the hygienic quality and the prevalence of the most common bacterial pathogens in fresh produce sold in retail markets in Sicily. A total of 125 samples of different types of vegetables were examined by standardized microbiological methods.

**Results:**

The aerobic mesophilic count ranged between 2 log and 7 log cfu g^−1^ and the *Enterobacteriaceae* counts between < 1 log and 6 log cfu g^−1^, with statistically significant differences between unprocessed and minimally processed products (*p* < 0.05). *Escherichia coli* was detected only in leaf vegetables at a concentration of 2 log - 3 log cfu g^−1^. Enterococci were found at a concentration of 2 log - 4 log cfu g^−1^. Coagulase positive Staphylococci and sulphite-reducing Clostridia were not detected in any sample. Three samples tested positive for *Listeria monocytogenes*, *Yersinia enterocolitica* and *Salmonella veneziana.*

**Conclusion:**

Our study provides updated data on the microbiological quality of retail vegetables and confirms the need to implement strategies to increase microbial safety of fresh produce.

## Background

Fresh produce plays an important role in the human diet because of its health-promoting nutritional characteristics [1]. Its antioxidants content in particular, is thought to be able to protect human cells from the attack of free radicals, which is in turn involved in the etiopathogenesis of most chronic diseases [2-4]. It is also hypothesized that vegetable antioxidants may bring further protective health effects through various mechanisms, acting as inducers of mechanisms related to cell maintenance, DNA repair and longevity [5-7]. This awareness has gradually resulted in the last years in a generalized shift of eating habits towards an increasing intake of fruits and vegetables. The data provided by the World Health Organization (WHO)/Food and Agriculture Organization (FAO) showed a 4.5% yearly increase in fruit and vegetable consumption between 1990 and 2004 [8]. Outbreaks of foodborne illnesses associated with the consumption of fresh produce are simultaneously increasing in frequency. In developed countries, changes in consumption patterns, raising numbers in elderly and immunocompromised consumers and the growing import of vegetables from countries with poor sanitary conditions have likely contributed to this epidemiological trend [9]. Moreover advanced diagnostic methods and surveillance systems have enhanced identification of fresh produce as sources of foodborne disease [10].

As a consequence, food safety of fresh produce is a matter of increasing concern; indeed, microbial contamination may occur during any of the steps in the farm-to-table continuum from environmental, animal or human sources [11-15].

Minimally processed vegetables (MPV) are processed to increase their functionality without changing their fresh properties. Preparation procedure generally includes processes such as washing, peeling, cutting, trimming and/or slicing [16]. *Salmonella* spp., *Shigella* spp., pathogenic *Escherichia coli*, *Listeria monocytogenes* and *Campylobacter* spp. are the most important vegetable-borne pathogens [11,15]. Data from the recent literature are inconsistent about the prevalence of these pathogens in vegetables, due to significant differences between studies in the sizes and place of sampling, local fresh produce type, seasonality and analytical methods [8]. Some authors report *Salmonella* spp. in less than 8% of the analyzed samples, *Campylobacter* spp. in 3.1% of lettuce, but *E. coli* O157 in up to 25% of cabbages and 19.5% of coriander and *L. monocytogenes* in up to 7% of cabbages, 22.7% of leafy vegetables and 20% of lettuce [8,17]. Other reports describe prevalence lower than 1-2% for *E. coli* O157, *Campylobacter* spp. and *Salmonella* spp. [18].

The aim of the study was to assess the hygienic quality and the prevalence of the most common pathogens in fresh produce, minimally processed vegetables (MPVs) and unprocessed vegetables (UVs), sold in retail markets in Sicily, Italy.

## Results and Discussion

### Microbial flora

The microbiological results of UVs are summarized in Table 1. The Aerobic Mesophilic Count (AMC) levels ranged between 2 log and 6 log cfu g^−1^ and the *Enterobacteriaceae* counts between 2 log and 6 log cfu g^−1^, except for the seed and bulb vegetable sample (<1 log cfu g^−1^). *Escherichia coli* was found only in leaf (lettuce) at a concentration of 2–3 log cfu g^−1^. Enterococci were found in leaf (lettuce), flower (cauliflowers) and fruit vegetables (marrow) at a concentration of 2–3 log cfu g^−1^.

**Table 1:** **Microbiological findings obtained from 85 samples of UVs (in log cfu g** ^**−1**^ **)**

**Vegetable**	**AMC (range)**	***Enterobacteriaceae*** **(range)**	***E. coli*** **(range)**	**Enterococci (range)**	**Clostridia**	**Coagulase positive staphylococci**
Seed vegetables	2 - 4	<1 - 3	<1	<1	<1	<1
Leaf vegetables	2 - 6	2 - 6	<1 - 3	<1 - 2	<1	<1
Fruit vegetables	3 - 6	2 - 3	<1	<1 - 3	<1	<1
Stem vegetables	2 - 6	2 - 3	<1	<1	<1	<1
Flower vegetables	4 - 5	2 - 3	<1	2	<1	<1
Root vegetables	4 - 5	2 - 3	<1	<1	<1	<1
Bulb vegetables	4 - 5	<1 -3	<1	<1	<1	<1

MPVs (Table 2) yielded AMC values between 5–7 log cfu g^−1^, except for the fruit vegetable samples (<1 log cfu g^−1^) (Table 2).

**Table 2:** **Microbiological findings obtained from 40 samples of MPVs (in log cfu g** ^**−1**^ **)**

**Vegetable**	**AMC (range)**	***Enterobacteriacea*** **(range)**	***E. coli*** **(range)**	**Enterococci (range)**	**Clostridia**	**Coagulase positive staphylococci**
Leaf vegetables	5 - 7	<1 - 5	<1 - 3	<1 - 3	<1	<1
Root vegetables	6 - 7	<1	<1	<1	<1	<1
Fruit vegetables	<1- 7	<1	<1	<1 - 4	<1	<1

*Enterobacteriaceae* and *E. coli* were detected in leaf vegetables (salad and spinach) and in one chicory sample. Enterococci were detected in salad samples and in a pumpkin sample at a concentration of 2–4 log cfu g^−1^. Coagulase positive Staphylococci and sulphite-reducing Clostridia were not detected in any sample.

The samples of the leaf vegetables had significantly higher AMC (*p* < 0.001) and *Enterobacteriaceae* (*p* = 0.01) counts than fruit vegetables. The differences of the *E. coli* counts between the two categories of vegetables were not statistically significant (*p* = 0.08), probably due to the lowest proportion of contaminated samples.

The colonies isolated from Violet Red Bile Glucose Agar proved to belong to the following bacterial species: *Klebsiella oxytoca, Pantoea* spp.*, Serratia odorifera, Serratia liquefaciens, Serratia ficaria, Raoultella terrigena, Rahnella aquatilis* and *Erwinia* spp*.*

Our findings showed that leaf vegetables had the highest bacterial counts among vegetable products, with particular reference to the mesophilic bacteria, *Enterobacteriaceae*, *E. coli* and Enterococci. Despite the high counts, no products reported visible signs of organoleptic alterations. Accordingly, Ragaert *et al*. [19] reported that evident organoleptic alterations occur in vegetables only when bacterial count is higher than 7 or 8 log cfu g^−1^.

High bacterial counts could likely be associated with the morphology of leaves which have a broad and rather rough surface. Indeed, both the large surface, easily coming in contact with the ground and the irrigation water, and its roughness facilitate the accumulation of dirt and adhesion of bacteria, as reported by some authors [10,20,21]. Our results agree with those reported by literature that identify salads as the most contaminated vegetable product. Seow *et al.* [10] in a study conducted on fresh vegetables and fruit samples, both UVs and MPVs, showed that samples of lettuce, along with sprouts, have the highest levels of mesophilic bacteria and coliforms. They reported mesophilic bacteria counts ranging from 3.4 to 7.3 log cfu g^−1^ and coliforms between 1.6 and 5.9 log cfu g^−1^, with 50% of samples containing more than 5 log cfu g^−1^ of coliforms. Allende *et al.* [22], while assessing the microbiological quality of red lettuce marketed in Spain, found 3.67 log cfu g^−1^ of coliforms. In Canada, Bohaychuk *et al.* [23] assessed the prevalence of *E. coli* in fresh vegetables produced in Alberta and found this organism in 8.2% of lettuce, spinach and carrots samples with counts ranging from 0.48 to 3.04 log MPN g^−1^. About UVs, it can be observed that Enterobacteria were present in almost all samples; counts higher than 2 log cfu g^−1^ were detected in 97.6% of the examined samples. However, this bacterial counts does not appear to be of special concern, as the identified species are commonly found in soil, water and in environments characterized by an excessive organic load. Thus, such microbial flora can be primarily attributed to an environmental source [10,24]. Regarding *E. coli* contamination*,* there was a low percentage of contaminated UVs samples (3.5%) with bacterial counts ranging from 2 to 3 log cfu g^−1^. Moreover, in our study, 5.0% of MPVs were contaminated by *E. coli*, but only one sample did not comply with the limits set by law [25], as the bacterial count was 4 log cfu g^−1^. Of interest, *E. coli* is included in the Regulation (EC) No 2073/2005 as the indicator of quality of hygienic processing for MPVs (pre-cut fruit and vegetables) [25]. Our data agree with those reported by literature, indicating low percentages of vegetable products contaminated with *E. coli* and with low counts. Abadias *et al.* [21] found *E. coli* in 7.1% of vegetable products of UVs and in 11.4% of products of MPVs, with only 0.8% of products exceeding 100 MPN g^−1^. Santos *et al.* [24] found 2.6% of contaminated samples (lettuce and spinach) with counts ranging between 1 and 2 log cfu g^−1^.

### Bacterial pathogens

Our results showed a total of three samples out of 125, one UV and two MPVs, yielding foodborne pathogens, in particular *Salmonella* spp., *L. monocytogenes* and *Y. enterocolitica*.

All samples tested negative for *Campylobacter* spp., *Shigella* spp. and *E. coli* O157.

*Salmonella veneziana* was detected in one sample of green salad (UVs). It yielded also an AMC of 6.98 log cfu g^−1^, an *Enterobacteriaceae* count of 5.73 log cfu g^−1^, an *E. coli* count of 3.95 log cfu g^−1^ and an Enterococci count of 2.07 log cfu g^−1^ (Table 3).

**Table 3:** **Characteristics of vegetables which tested positive for foodborne pathogens**

**Vegetable**	**Type**	**Microrganism**	**AMC log cfu g** ^**−1**^	***Enterobacteriaceae*** **log cfu g** ^**−1**^	***E. coli*** **log cfu g** ^**−1**^	**Enterococci log cfu g** ^**−1**^
Pumpkin	MPV	*L. monocytogenes*	6.47	<1	<1	<1
		*L. innocua*				
Spinach	MPV	*Y. enterocolitica*	6.95	4.90	<1	<1
		*A. hydrofila*				
Green salad	UV	*Salmonella* spp.	6.98	5.73	3.95	2.07

*Listeria monocytogenes* serotype 4b was detected in association with *L. innocua* in a pumpkin sample. This was a minimally processed vegetable sample and the quantitative analysis yielded a concentration of *Listeria* less than 1 log cfu g^−1^. This sample had also an AMC of 6.47 log cfu g^−1^ (Table 3).

*Yersinia enterocolitica* O:3 was detected in a MPV spinach sample which yielded also *Aeromonas hydrofila.* Moreover, this sample showed an AMC of 6.95 log cfu g^−1^ and an *Enterobacteriaceae* count of 4.90 log cfu g^−1^ (Table 3).

Literature data are conflicting about the prevalence of pathogens in vegetables. Studies on UV fresh vegetables in USA, United Kingdom, and Malaysia showed a highly variable prevalence of *Salmonella* contamination, with values between 0% and 35%, respectively [18,26,27]. During 2005–2006, in Spain, Abadias *et al.* [21] detected *Salmonella* spp. and *L. monocytogenes,* respectively, in 1.3% and 0.7% of their samples. Furthermore, Sant’Ana *et al.* [28] studied the prevalence of *Salmonella* spp. in MPVs and found that 0.8% of the samples was contaminated by serotypes *Enteritidis* and *Typhimurium*. In Norway, Johannessen *et al.* [29] isolated *L. monocytogenes* and *Y. enterocolitica*, respectively, in 0.3% and 3% of the samples. Santos *et al.* [24] tested MPV samples for pathogens and found *A. hydrophila* in 11 samples (7.3%), *L. monocytogenes* in one sample of spinach (0.6%) and *L. innocua* in a further sample of spinach and salad (1.3%). Pathogen contamination in MPVs suggests failures in risk assessment and management systems in processing facilities, where contamination sources may be very heterogeneous: soil, irrigation waters and processing environment. Moreover washing and disinfection may not provide a complete removal of pathogens from MPVs. It is, indeed, of the uttermost importance to accurately follow Good Manufacturing Practices (GMP) through the entire production cycle (from primary production to distribution), in order to consistently meet food safety objectives.

## Conclusion

Community regulation about the hygienic quality of vegetables is quite recent. Regulation (EC) 2073/2005 and subsequent modifications, has established microbiological limits for some types of vegetable products. In particular for ready-to-eat pre-cut fruits and vegetables, *E. coli* and *L. monocytogenes/Salmonella* spp. were defined as microbiological criteria of process hygiene and food safety, respectively. No further specific regulation is in place in EU for other types of vegetable products, such as UVs, or other pathogen microorganisms (e.g. *Yersinia* spp.*, Campylobacter* spp.). Even though there are worldwide reports of outbreaks associated with the consumption of vegetable products, data concerning the microbial contamination level of these foodstuffs are still few and discrepant; in particular this lack of knowledge affects most MPV and UV vegetables.

This work is to some extent a preliminary investigation which will allow for targeting specific groups of products with a higher risk profile in the next future.

## Methods

### Vegetable samples

One hundred twenty-five vegetable samples, (40 MPVs and 85 UVs), were collected from four supermarkets and two greengrocer’s shops (Table 4) in Palermo district.

**Table 4:** **Number and type of vegetable samples under analysis**

Vegetable*	**No. of samples**	**No. of UV samples**	**No. of MPV samples**
Leaf vegetables	58	28	30
Bulb vegetables	3	3	/
Root vegetables	13	7	6
Fruit vegetables	37	33	4
Flower vegetables	4	4	/
Stem vegetables	7	7	/
Seed vegetables	3	3	/
Total	125	85	40

*Leaf vegetables (lettuce, chicory, spinach), bulb vegetables (garlic), root vegetables (carrot), fruit vegetables (aubergine, tomato, marrow, pumpkin), flower vegetables (cauliflower), stem vegetables (fennel, celery), seed vegetables (sesame).

These variety of places allowed products commonly available to consumers to be sampled, thus making results more representative.

Sampling was carried out by randomly taking 250–300 g of sample from each box, according to the Regulation (EC) No 333/2007 [30]. The samples of MPVs were bought within 1–2 days from their packaging. All samples were subjected to microbiological analysis after an inspective check to evaluate the following features: for UVs, freshness and absence of spoilage signs; for MPVs, labeling information and presence of extraneous materials into the wrapping.

The samples were transferred to the laboratory in cooler boxes at temperature between 1°C and 8°C and the microbiological assays were performed within 24 hrs since the sample collection.

### Enumeration of bacteria

Each sample was analyzed for Aerobic Mesophilic Count (AMC), *Enterobacteriaceae*, *E. coli* β-glucuronidase positive, coagulase positive Staphylococci, sulphite-reducing anaerobic organisms, Enterococci and *L. monocytogenes*. Microbial analyses were carried out using the standard methodologies described in Table 5.

**Table 5:** **Standard methods used for microbial analyses**

**Determination**	**Methodology**	**Medium, temperature and incubation time**
Aerobic Mesophilic Count (AMC)	ISO 4833:2003	Plate Count Agar, 30°C, 72 hrs
*Enterobacteriaceae*	ISO 21528–2:2004	Violet Red Bile Glucose Agar, 37°C, 24 hrs
*E. coli* β-glucuronidase positive	ISO 16649–2:2010	Tryptone Bile Glucuronide Agar, 44°C, 24 hrs
Coagulase positive staphylococci	ISO 6888–1:1999 Amend. 1:2003	Baird Parker Agar, 37°C, 24–48 hrs
Sulphite-reducing anaerobic organisms	ISO 15213:2003	Iron Sulphite Agar, 37°C, 24 hrs
*L. monocytogenes*	ISO 11290–2:1998	ALOA Agar, 37°C, 24–48 hrs

Thirty g of each sample were weighed into sterile stomacher bags and homogenized with 270 ml Saline Peptone solution (NaCl 8.5 g l^−1^, Peptone l.0 g l^−1^) in a stomacher (Type 400; Seward London, UK). Decimal dilutions were prepared with the same diluent and 1 ml of each was used as inoculum. Results were reported in terms of colony forming units (cfu g^−1^).

A laboratory internal method (Rapid Enterococcus Agar, 44°C, 48 hrs, followed by catalase and esculin hydrolysis test on the suspected colonies) (Biorad) was used for Enterococci.

Representative colonies of all discernible morphologies were picked up from the Violet Red Bile Glucose Agar plates, subcultured and biochemically identified by API 20E (bioMerieux, Marcy-l’Etoile, France).

### Isolation of foodborne pathogens

Twenty five g of each sample were weighed into sterile stomacher bags and homogenized with 225 ml of enrichment broth for each pathogen.

*Shigella* spp. detection was performed by the ISO 21567:2004 method consisting of a first enrichment step in *Shigella* Broth with novobiocin at 41.5°C for 16–20 hrs, followed by subculture in MacConkey Agar, Xylose Lysine Deoxycholate (XLD) Agar and Hektoen Enteric Agar at 37°C for 20–24 hrs.

*Yersinia enterocolitica* detection was performed by the ISO 10273:2003 method with a selective enrichment in Pepton Sorbitol Bile Salts (PSB, Biolife) at 25°C for 5 days and an additional selective enrichment in Irgasan Ticarcillin (ITC, Biolife) Broth at 25°C for 48 hrs. The PBS broth was inoculated in Cefsulodin Irgasan Novobiocin Agar, while ITC Broth was streaked onto *Salmonella-Shigella* Agar with Sodium Deoxycholate and Calcium Chloride (Biolife, Sigma). All the plates were incubated at 30°C for 24 hrs.

*Campylobacter* spp. detection was performed by the ISO 10272–1:2006 method with a selective enrichment in Bolton Broth at 37°C for 4–6 hrs and then at 41.5°C for 48 hrs microaerobically. The Bolton Broth was inoculated in modified *Campylobacter* Charcoal Differential Agar (Biolife) and Skirrow Agar at 41.5°C for 48 hrs microaerobically.

Detection of *Salmonella* spp., *L. monocytogenes* and *E. coli* O157 was carried out by an enzyme linked fluorescent assay (ELFA) in an automatic system VIDAS (bioMerieux, Marcy-l’Etoile, France). In particular, the following methods were used:

- for *Salmonella* spp., the AFNOR BIO 12/23-05/07 method including a pre-enrichment step in Buffered Peptone Water at 37°C for 16–20 hrs and a subsequent step performed by VIDAS Immuno-Concentration *Salmonella* II (ICS2). The samples which tested positive were then confirmed by subculturing them in XLD Agar at 37°C for 24 ± 3 hrs;
- for *L. monocytogenes*, the AFNOR BIO 12/11-03/04 method was performed with Half Fraser broth at 30°C for 24–26 hrs and then Fraser Broth (FB) at 37°C for 24–26 hrs. One portion of the FB culture was then used for the *L. monocytogenes* VIDAS test (LMO2). The samples which tested positive were then confirmed by subculturing them in *Listeria* Aloa Agar (ALOA) (Biolife) and *Listeria* Oxford Agar at 37°C for 24 ± 3 hrs;
- for *E. coli* O157, the AFNOR BIO 12/8-07/00 method was used with a first step in Tryptone Soya Broth with novobiocin, incubated at 41.5°C for 6–7 hrs, and a second step in MacConkey Broth with cefixime-potassium tellurite, incubated at 37°C for 18 hrs. After heating at 95-100°C, an aliquot was used for VIDAS (ECO).

All culture media were from OXOID except otherwise stated.

### Biochemical and serological identification

The suspected *Salmonella* spp. colonies were subcultured for purity and identified by the Biolog automatic system (Biolog Inc., Hayward, CA). All suspected *Listeria* spp. colonies were submitted to catalase and β-hemolysis test and definitively identified by the Biolog automatic system.

*Listeria monocytogenes* isolates were subjected to serotyping by specific antisera (Denka Seiken, Tokio, Japan). The suspected *Yersinia* spp. colonies were submitted to oxidase, urease, indole test and definitively identified by API 20E (bioMerieux, Marcy-l’Etoile, France).

Serotyping of *Salmonella*, *L. monocytogenes* and *Y. enterocolitica* isolates was carried out at the Regional Reference Centre for Enteric pathogens, University of Palermo, Italy, by using commercial antisera (Staten Serum Institut, Denmark, and Denka-Seiken, Japan).

### Statistical analysis

Quantitative values were categorized in two classes (equal/higher *vs* lower than the median value for AMC and *Enterobacteriaceae* and detected *vs* undetected for *E. coli*). Association between these classes and the most represented type of vegetables was assessed by contingency tables. Statistical significance was calculated by the chi-square test or the Fisher’s exact test, when appropriate. *p* value less than 0.05 was considered statistically significant.

## Abbreviations

ALOA: *Listeria* Aloa agar
AMC: Aerobic mesophilic count
ELFA: Enzyme linked fluorescent assay
FAO: Food and Agriculture Organization
FB: Fraser broth
GMP: Good Manufacturing Practices
ITC: Irgasan Ticarcillin
MPVs: Minimally processed vegetables
PSB: Pepton Sorbitol Bile Salts
UVs: Unprocessed vegetables
WHO: World Health Organization
XLD: Xylose Lysine Deoxycholate
